# Postoperative Changes in *In Vivo* Measured Friction in Total Hip Joint Prosthesis during Walking

**DOI:** 10.1371/journal.pone.0120438

**Published:** 2015-03-25

**Authors:** Philipp Damm, Alwina Bender, Georg Bergmann

**Affiliations:** Julius Wolff Institute, Charité—Universitaetsmedizin Berlin, Berlin, Germany; VIT University, INDIA

## Abstract

Loosening of the artificial cup and inlay is the most common reasons for total hip replacement failures. Polyethylene wear and aseptic loosening are frequent reasons. Furthermore, over the past few decades, the population of patients receiving total hip replacements has become younger and more active. Hence, a higher level of activity may include an increased risk of implant loosening as a result of friction-induced wear. In this study, an instrumented hip implant was used to measure the contact forces and friction moments *in vivo* during walking. Subsequently, the three-dimensional coefficient of friction *in vivo* was calculated over the whole gait cycle. Measurements were collected from ten subjects at several time points between three and twelve months postoperative. No significant change in the average resultant contact force was observed between three and twelve months postoperative. In contrast, a significant decrease of up to 47% was observed in the friction moment. The coefficient of friction also decreased over postoperative time on average. These changes may be caused by ‘running-in’ effects of the gliding components or by the improved lubricating properties of the synovia. Because the walking velocity and contact forces were found to be nearly constant during the observed period, the decrease in friction moment suggests an increase in fluid viscosity. The peak values of the contact force individually varied by 32%-44%. The friction moment individually differed much more, by 110%-129% at three and up to 451% at twelve months postoperative. The maximum coefficient of friction showed the highest individual variability, about 100% at three and up to 914% at twelve months after surgery. These individual variations in the friction parameters were most likely due to different ‘running-in’ effects that were influenced by the individual activity levels and synovia properties.

## Introduction

Loosening of the artificial cup and inlay is the most common reason for the failure of total hip replacements [1–3]. Polyethylene (PE) wear and aseptic loosening are frequent reasons for revisions of total hip joint prostheses (THP), account for 26% and 48% of all revisions, respectively [1,4]. One study has indicated that 30% to 40% of all THP revisions require a change of cup or inlay [5], thus making friction in the joint one of the main parameters affecting the life span of total hip prostheses. THP patients have become younger and more active over the past few decades [6,7]. This higher level of activity has resulted in increased wear rates and, consequently, requires earlier revision of THP [8,9].

To determine the parameters of friction in THP, several *in vitro* studies under different test conditions have been published, using new or explanted prostheses and different head diameters and lubricants [10–21]. In most studies, the coefficient of friction (μ) was determined by a simple pendulum test, which showed that the friction in the THP was primarily influenced by the material of the sliding partners and the lubrication regime.

Previous studies with instrumented implants measured the contact forces in THP *in vivo* [22–26]. However, in these studies, it was not possible to determine the friction within the implant, with the exception of one study that reported the friction-induced temperature increase during walking [27]. In that publication, it was postulated that the temperature in the THP was mainly caused by the friction parameters between the sliding partners and the lubricating properties of the synovia. Peak temperatures up to 43°C were measured after one hour of walking. Because bone tissues may already be damaged at temperatures exceeding 43°C [28], it can be assumed that repeated high implant temperatures are a risk factor for the long-term stability of the THP.

Using a newly developed instrumented hip implant [29], we were able to measure the joint friction *in vivo* for the first time. Initial results were reported for three months postoperatively (pOP) [30], and it was shown that the *in vivo* coefficient of friction is similar to the *in vitro* value obtained during the extension phase of the hip joint. However, during the flexion phase, μ strongly differs from the *in vitro* data. The maximum *in vivo* values of μ, calculated from the contact forces and friction moments, suggest a ‘dry’ friction during the hip flexion phase of walking.

The aim of this study was to measure the changes of contact forces, friction moments and coefficients of friction between three and twelve months pOP. We hypothesized that μ would decline due to ‘running-in’ effects and that both, friction moments and μ, would show great individual variability, depending on the synovial properties.

## Methods

### Variables

F_res_ Resultant hip joint contact force

F_res_ Vector of F_res_

M_res_ Resultant friction moment

M_res_ Vector of M_res_

F_Friction_ Friction force

F_Friction_ Vector of F_Friction_

R Radius of implant head (16 mm)

R Vector from origin of coordinates to contact point P

D Vector from origin of coordinates to vector H

H Vector vertical to M_res_, pointing to P

μ Coefficient of friction

HS Ipsilateral heel strike

CTO Contralateral toe off

CHS Contralateral heel strike

TO Ipsilateral toe off

XPE Cross-linked polyethylene

### Measurement equipment

Joint forces and friction moments were measured *in vivo* using instrumented hip implants. The prosthesis (CTW, Merete Medical, Berlin, Germany) is based on a clinically successful implant with a titanium stem, a 32 mm Al_2_O_3_ ceramic head and a XPE inlay. A telemetry circuit, six-strain gauges and an induction coil are arranged in the hollow neck. The implant is powered inductively by a coil around the hip joint. The strain gauge signals are transferred to the external receiver at radio frequency via an antenna inside of the implant head [31]. The telemetric load signals and the patient movements are recorded simultaneously on video. Detailed descriptions of the implant [29] and the external measurement system [31,32] have previously been published.

Using the six strain gauge signals, the three force and three moment components acting on the implant head are calculated with an accuracy of 1–2%. The femur-based coordinate system [33] is located in the head center of a right-sided implant; data from left-sided implants are mirrored. Positive forces F_x_, F_y_, and F_z_ act in the lateral, anterior, and superior directions; the measured friction moments (M_x_, M_y_ and M_z_) turn right around the positive coordinate axes. The resultant contact force F_res_ and the resultant friction moment M_res_ are calculated from their three components. Because the force component in direction of the femoral axis always acts downwards, F_res_ points toward the center of the implant head.

### Patients and measurements

The study was approved by the ethical committee (EA2/057/09) and registered with the ‘German Clinical Trials Register’ (DRKS00000563). Ten patients (8m/2f) with hip osteoarthritis gave written informed consent to participate in the study and have their images published (Table 1).

**Table 1:** Patient data and walking speeds.

Subject pOP	Age [years]	Sex	Bodyweight [N]	Gait Velocity [m/s]	
	12 months pOP		12 months pOP	3 months pOP	12 months pOP
H1L	56	male	760	1.0	1.1
H2R	62	male	767	1.0	1.1
H3L	60	male	983	0.8	1.0
H4L	51	male	796	1.0	1.0
H5L	63	female	855	0.9	1.2
H6R	69	male	815	1.1	1.1
H7R	53	male	916	1.1	1.3
H8L	56	male	836	1.1	1.1
H9L	55	male	1197	1.1	1.2
H10R	54	female	995	0.9	1.2
Average	58	-	892	1.0	1.1

Measurements were taken during level walking at several time points (Table 2) between three and thirteen months pOP. Selected trials of each investigated subject are also shown and can be downloaded at the public data base www.OrthoLoad.com.

**Table 2:** Number of averaged steps at postoperative months 1 to 13.

Patient	Month pOP												
	1	2	3	4	5	6	7	8	9	10	11	12	13
H1L	-	-	67	-	-	-	71	-	-	56	-	-	67
H2R	-	32	49	-	-	65	88	-	-	-	86	52	-
H3L	-	32	82	72	-	-	-	-	45	-	-	105	-
H4L	30	98	53	-	51	-	48	-	-	36	-	63	-
H5L	-	41	69	-	58	-	-	-	-	69	-	41	-
H6R	-	54	71	-	65	-	51	-	-	83	-	44	-
H7R	-	93	96	-	60	46	-	66	-	77	-	45	-
H8L	-	31	50	-	59	-	-	-	-	58	-	53	-
H9L	43	85	84	-	43	60	-	-	-	-	-	-	83
H10R	-	104	30	-	91	-	-	39	-	-	-	28	-
≈ Months pOP	“3” (3)			“6” (5 to 6)			“9” (8 to 10)				“12” (12 to 13)		
Average	10 Subjects			9 Subjects			8 Subjects				10 Subjects		

All available data were used to calculate postoperative trends in single subjects.

### Data evaluation

All forces and moments are reported as a percentage of patient bodyweight (%BW and %BWm, respectively). Average force- and moment-time patterns were calculated separately for each subject and measurement day using a ‘time warping’ method [34]. This averaging procedure was first performed on repeated trials of the single subjects. The obtained load patterns of all subjects were then averaged again, leading to data which are typical for an ‘average’ subject. All data were analyzed for the time points of three, six, nine and twelve months pOP (Table 2).

Unless stated otherwise, all presented data refers to the obtained ‘average’ subject. Because errors between the single trials were minimized over all loading cycles, the peak values of the average curves can slightly deviate from the averaged numerical values at the 1^st^ and 2^nd^ peak.

Changes of the measured peak values over the pOP time were analyzed for each subject separately and for the ‘average’ subject (Tables 3 and 4). The changes over time were analyzed individually using the Mann-Whitney-U test and across all subjects using the Wilcoxon test. Furthermore a regression analysis was performed, for each subject separately and for the average subject, to determine the correlation between the measured peak values of F_res_, M_res_ and μ (y) and the pOP time in months (m), using the logarithmic relationship

y = a + b*ln(m) (1)

The coefficient of correlation (R²) and the residual-standard-error (RSE) were calculated and statistically analyzed with a t-test. For all statistical calculations a type-I-error level was defined with α = 0.05.

**Table 3:** Mean values at 3 and 12 months post operative of the ‘average’ subject.

value	unit	3 months		12 months	
		Mean	STD	Mean	STD
Fres1	%BW	*255*	*10*	*252*	*12*
Fres2	%BW	*238*	*9*	*245*	*8*
Mres1	%BWm	*0.174*	*0.019*	*0.107*	*0.017*
Mres2	%BWm	*0.234*	*0.020*	*0.174*	*0.020*
μ1	-	*0.045*	*0.005*	*0.028*	*0.004*
μ2	-	*0.062*	*0.005*	*0.047*	*0.005*
μmax	-	*0.175*	*0.020*	*0.179*	*0.036*

Arithmetic means and standard deviation (STD) of the contact force (F_res_), friction moment (M_res_) and coefficient of friction (μ).

**Table 4:** Load and friction changes.

Subject	*F*_*res1*_	*p(F_res1_)*	*F*_*res2*_	*p(F_res2_)*	*M*_*res1*_	*p(M_res1_)*	*M*_*res2*_	*p(M_res2_)*	*μ*_*1*_	*p(μ_1_)*	*μ*_*2*_	*p(μ_2_)*	*μ*_*max*_	*p(μ_max_)*
H1L	6	<0.001	4	<0.001	-37	<0.001	-9	<0.001	-41	<0.001	-14	<0.001	-13	<0.001
H2R	1	0.306	8	<0.001	-72	<0.001	-36	<0.001	-72	<0.001	-44	<0.001	-15	0.029
H3L	-4	<0.001	2	<0.001	-51	<0.001	-2	<0.001	-48	<0.001	-6	<0.001	22	<0.001
H4L	4	<0.001	3	<0.001	-44	<0.001	-52	<0.001	-46	<0.001	-53	<0.001	-26	<0.001
H5L	-1	0.467	3	<0.001	-45	<0.001	-34	<0.001	-44	<0.001	-34	<0.001	-17	<0.001
H6R	5	<0.001	13	<0.001	-35	<0.001	-34	<0.001	-38	<0.001	-42	<0.001	-27	<0.001
H7R	0	0.903	6	<0.001	-38	<0.001	-28	<0.001	-36	<0.001	-32	<0.001	57	<0.001
H8L	-13	<0.001	-2	0.039	-67	<0.001	-60	<0.001	-56	<0.001	-59	<0.001	-12	<0.001
H9L	-3	<0.001	-1	0.083	-33	<0.001	-49	<0.001	-37	<0.001	-48	<0.001	-42	<0.001
H10R	-1	0.083	-8	<0.001	13	<0.001	30	<0.001	18	<0.001	48	<0.001	101	<0.001
AverageSubject H	-1	0.863	4	0.730	-47	0.004	-34	0.003	-46	0.002	-37	0.004	-8	0.605

Individual and average change [%] of peak joint contact forces, friction moments and coefficients of friction from 3 to 12 months postoperative. Arithmetic mean values were calculated without the data from subject H10R. Tests: Mann-Whitney-U. (individual subjects) respectively Wilcoxon test (‘average’ subject).

### Coefficient of friction

**Fig 1:**
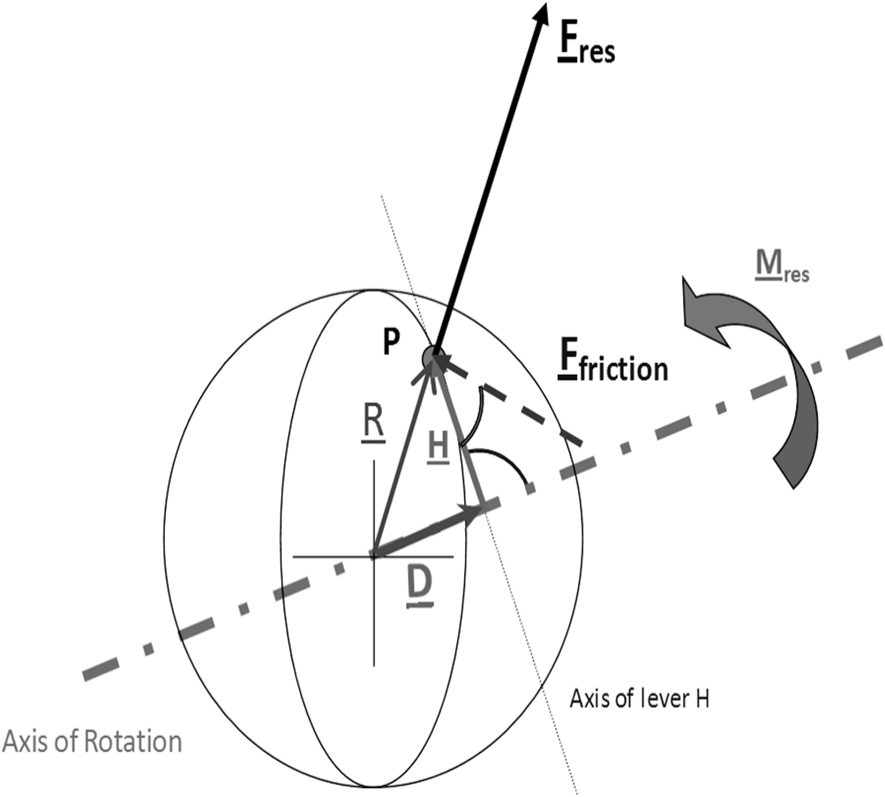
Model for calculating the 3D coefficient of friction μ. In reality, the component F_z_ is measured negatively, and the direction of F_res_ is thus towards the ball surface

In the following equations, the underlined symbols are vectors; all others are scalar values. In the Coulomb approach, the friction force F_Friction_ between the cup and ball is determined using the resultant contact force F_res_ and the coefficient of friction μ (Fig. 1):

$${\text{F}}_{\text{Friction}}{\;\text{= μ * F}}_{\text{res}}$$ (2)

The ball turns around the axis of M_res_. H is the lever arm between F_Friction_ and the axis of M_res_. Because F_Friction_ counteracts the moment M_res_, we obtain

$${\text{M}}_{\text{res}}{\text{ = H * F}}_{\text{Friction}}$$ (3)

(2) and (3) deliver

$${\text{μ = M}}_{\text{res}}\;\text{/}\;\left({\text{H * F}}_{\text{res}}\right)$$ (4)

R is the radius of the ball and points in direction of F_res_:

$$\underline{R}=\;R\;*{\underline{F}}_{res}/\;F_{res}$$ (5)

H can be substituted by

$$\underline{H}=\underline{R}\;-\;\underline{D}$$ (6)

with D being the orthogonal projection of R on M_res_:

$$\underline{D}=\;R\;*\;cos(\underline{R},{\underline{M}}_{res})\;*{\underline{M}}_{res}/\;M_{res}$$ (7)

Applying (5) and (7), equation (6) becomes

$$\underline{H}=\;R\;*\;[{\underline{F}}_{res}/\;F_{res}\;-\;cos(\underline{R},M_{res})\;*\;({\underline{M}}_{res}/\;M_{res})]$$ (8)

with

$$cos(\underline{R},{\underline{M}}_{res})\;=\;cos({\underline{F}}_{res},{\underline{M}}_{res})\;=\;<{\underline{F}}_{res},{\underline{M}}_{res}>\;/\;\left(F_{res}*\;M_{res}\right)$$ (9)

because equation (5) holds true.

To obtain μ with an accuracy of 5%, only joint forces F_res_ ≥ 25%BW and moments M_res_ ≥ 0.02%BWm were included in the analysis.

### Gait velocity

The gait velocity was determined individually at three months pOP using the video clips, as described elsewhere [30]. At twelve months pOP, the velocity was determined individually using simultaneously measured 3D gait data (Vicon Nexus, Vicon Motion Systems Ltd., UK). The distance travelled during one walking trial was measured between the first and last HS of the ipsilateral leg for each walking trial separately. It was divided by the elapsed time and the obtained speed was subsequently averaged over all trials.

## Results

**Fig 2:**
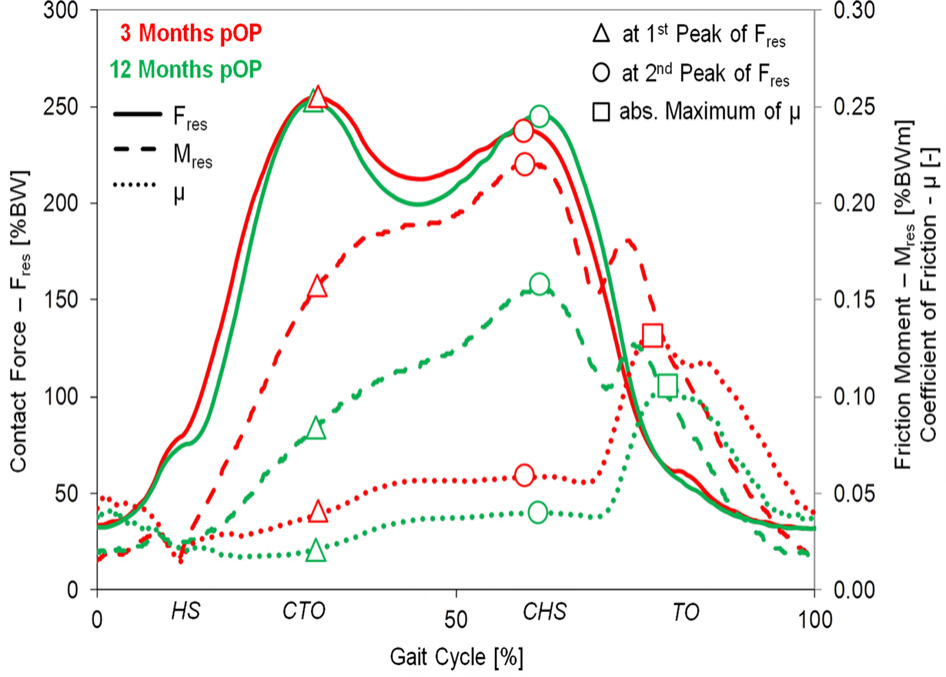
Postoperative changes of loads and friction. Average time courses of F_res_, M_res_ and μ during one gait cycle at three and twelve months pOP. The indicated values M_res1_, M_res2_, μ_1_, μ_2_ and μ_max_ were determined at the instants of the peak forces F_res1_ and F_res2_.

During gait, the resultant joint force F_res_ had two extreme values (Fig. 2), labeled F_res1_ and F_res2_. At the instants of these peak forces the friction moments M_res1_ and M_res2_ were determined. The coefficient of friction μ was calculated throughout the whole gait cycle and the values μ_1_ and μ_2_ at the instants of F_res1_ and F_res2_ plus the absolute maximum value μ_max_ were analyzed.

### Joint contact force

The resultant joint force F_res_ was calculated using the measured force components in lateral, anterior, and superior directions. In Fig. 2, the patterns of F_res_ (bold lines) of the ‘average’ subject are shown for two different time points, three and twelve months pOP. Both patterns are similar and show two typical peak values. The first maximum (F_res1_) occurs at the time of contralateral toe off (CTO), and the second maximum (F_res2_) at the contralateral heel strike (CHS).

**Fig 3:**
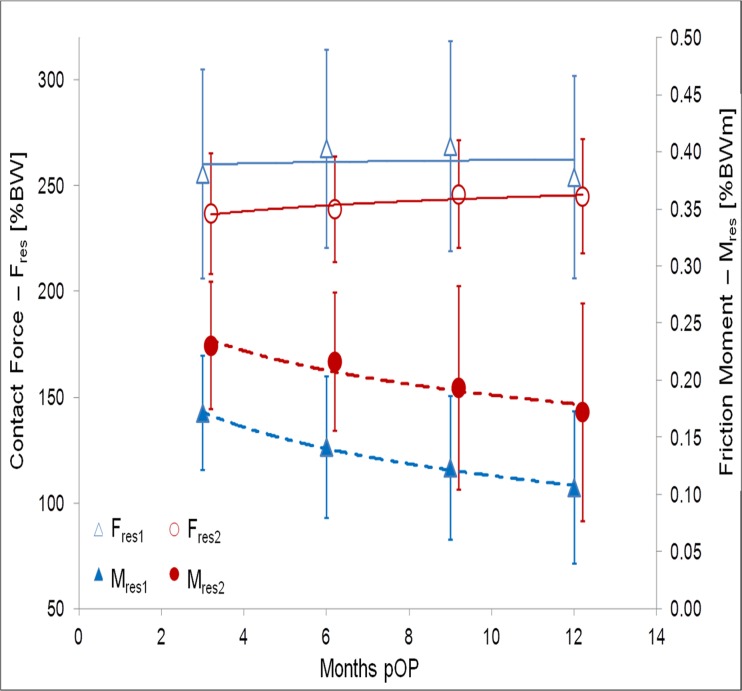
Postoperative changes of contact forces and friction moments. Average and standard deviations.

**Fig 4:**
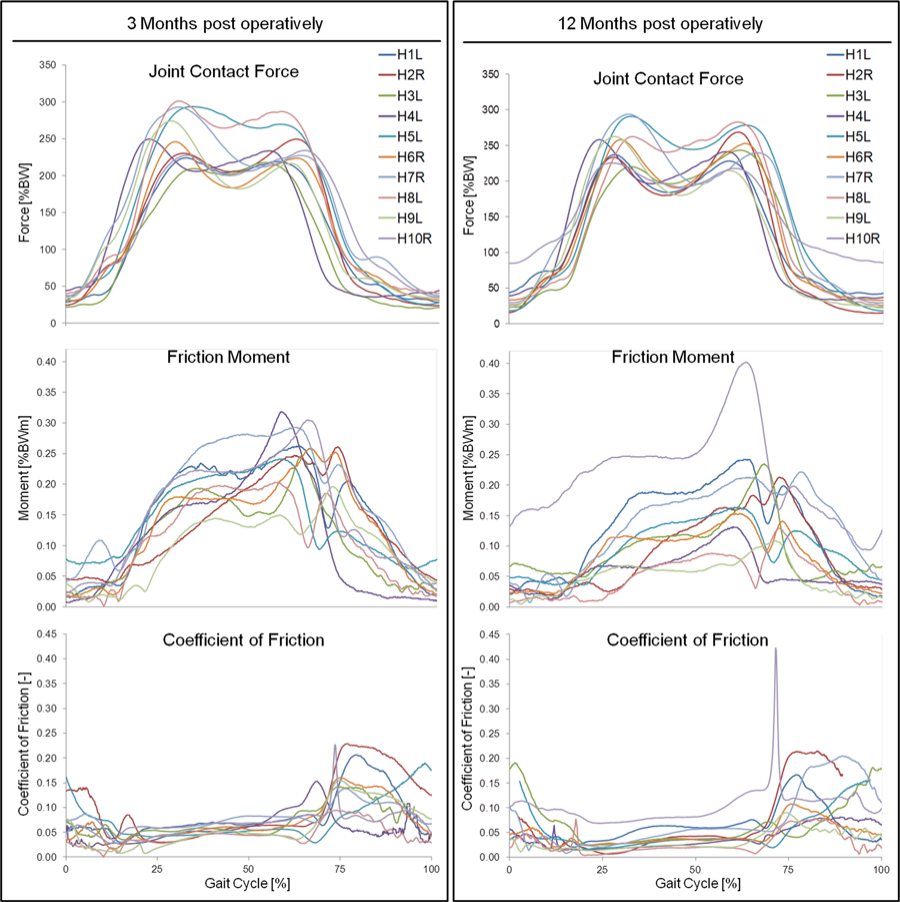
Individual contact forces, friction moments and coefficients of friction. Load pattern during one gait cycle at three (left) and twelve (right) months postoperatively.

At three months pOP, peak values of 255%BW (F_res1_) and 238%BW (F_res2_) were determined for the ‘average’ subject (Figs. 2 and 3, Table 3). The values of F_res1_ individually ranged between 209%BW (H3L) and 301%BW (H8L) (Fig. 4), which is a variation by 44% F_res2_ laid between 217%BW (H9L) and 287 (H8L); it had a variation of 32%.

At twelve months pOP (Figs. 2 and 3, Table 3), peak values of 252%BW (F_res1_) and 245%BW (F_res2_) were determined for the ‘average’ subject. F_res1_ individually varied (Fig. 4) between 220%BW (H3L) and 294%BW (H7R) by 34%. F_res2_ ranged between 215%BW (H9L) and 283%BW (H8L) with a variation of 32%.

Over the three- to twelve-month pOP period (Figs. 2, 3 and 4), F_res1_ declined by -1% on average (Table 4). It increased in four subjects, but decreased in five subjects and remained constant in one (H7R). The individual changes laid between +6% (H1L) and -13% (H8L). The change of F_res2_ during the pOP time was +4%. F_res2_ increased in seven and decreased in three subjects (Table 4). The individual changes laid between -8% (H10R) and +13% (H6R). For the ‘average’ subject a logarithmic correlation was determined between the pOP month and the peak values F_res1_ and F_res2_ (Fig. 3) with R² = 0.03 and R² = 0.68 (Table 5).

**Table 5:** Average postoperative trends of the contact force (F_res_), friction moment (M_res_) and coefficient of friction (μ).

Average	R²	RSE	a	p(a)	b	p(b)
Fres1	*0.023*	*7.657*	*265*	*0.002*	*-1.592*	*0.849*
Fres2	*0.679*	*2.831*	*232*	*0.001*	*5.597*	*0.176*
Mres1	*0.994*	*0.003*	*0.225*	*0.001*	*-0.048*	*0.003*
Mres2	*0.917*	*0.009*	*0.281*	*0.004*	*-0.042*	*0.043*
μ1	*0.992*	*0.001*	*0.059*	*0.001*	*-0.013*	*0.004*
μ2	*0.993*	*0.001*	*0.073*	*<0.001*	*-0.011*	*0.004*
μmax	*0.251*	*0.009*	*0.166*	*0.010*	*0.007*	*0.499*

Logarithmic regression functions y = a + b*ln(m) with m = postoperative month, the residual-standard-error (RSE) and the p-values for the intercept a and the slope b (t-test)

### Friction moment

During walking, M_res_ increased in all patients between heel strike (HS) and CHS (Fig. 2, dashed lines), with a first maximum around CHS. The local minimum of the M_res_ curve between CHS and TO represents the instant when the rotational direction of the joint changes from extension to flexion. It is followed by a second, smaller maximum during the flexion phase, shortly before TO.

At three months pOP, friction moments of 0.174%BWm (M_res1_) and 0.234%BWm (M_res2_) were determined for the ‘average’ subject (Figs. 2 and 3, Table 3). After the local minimum, a smaller second maximum of 0.186%BWm was determined during the flexion phase of the gait cycle. M_res1_ individually ranged between 0.096%BWm (H9L) and 0.220%BWm (H7R), a variation by 129%. M_res2_ laid between 0.137%BWm (H9L) and 0.301%BWm (H10R) (Fig. 4) and varied by 119%. The smaller second maximum ranged from 0.124%BWm (H5L) to 0.261%BWm (H2R) with a variation of 110%.

At twelve months pOP (Figs. 2 and 3, Table 3), average friction moments of 0.107%BWm (M_res1_) and 0.174%BWm (M_res2_) were found. M_res1_ ranged between 0.027%BWm (H2R) and 0.143%BWm (H1L); it highly varied by 430% (Fig. 4). M_res2_ laid between 0.069%BWm (H9L) and 0.402%BWm (H10R), which is an even higher variation of 451%. The second smaller maximum had an average of 0.130%BWm. It ranged from 0.045%BWm (H4L) to 0.247%BWm (H10R) and varied by 449%.

From three to twelve months pOP, M_res1_ decreased significant on average by -47% (Table 4). But this change was individually very different, with values between +13% (H10R) and -72% (H2R). M_res2_ sunk on average by -34%, with individual changes between +30% (H10R) and -60% (H8L). The smaller second maximum of M_res_ was decreased by -28%. With exception of H10R, M_res1_ and M_res2_ decreased in all subjects over the first twelve months pOP.

A logarithmic correlation was observed between the pOP month and the peak values M_res1_ and M_res2_ (Fig. 3) with R² = 0.994 and R² = 0.917 for the ‘average’ subject (Table 5). The slope of M_res_ from HS to CTO decreased by approximately -50% during the pOP time.

### Coefficient of friction

The coefficient of friction (μ) increased throughout the whole load phase from HS to TO (Fig. 2, dotted lines). This increase was approximately linear during the stance phase between CTO and CHS. Shortly after the CHS, at approximately 70% of the gait cycle, μ increased sharply and always reached its absolute maximum (μ_max_) shortly before TO.

At three months pOP (Fig. 2, Table 3), values of μ_1_ = 0.045, μ_2_ = 0.062 and μ_max_ = 0.175 were determined for the ‘average’ subject. As with F_res_ and M_res_, the coefficient of friction individually varied much (Fig. 4). μ_1_ ranged from 0.032 (H8L) to 0.063 (H1L), and varied by 97%. μ_2_ laid between 0.04 (H9L) and 0.081 (H10R), which is a variation by 103%. μ_max_ had values between 0.095 (H8L) and 0.229 (H2R); it varied by 141%.

At twelve months pOP (Fig. 2, Table 3), values of μ_1_ = 0.028, μ_2_ = 0.047 and μ_max_ = 0.179 were determined for the ‘average’ subject (Fig. 2). μ_1_ varied extremely between 0.007 (H2R) to 0.071 (H10R), which is a variation of 914%. μ_2_ was individually less different; it had values between 0.02 (H9L) to 0.116 (H10R) and thus varied by 480%. μ_max_ ranged from 0.079 (H8L) to 0.442 (H10R) with a variation of 459%.

From three to twelve months pOP (Figs. 2, 3 and 4), the coefficient of friction decreased on average (Table 4). The reduction of μ_1_ was -34%, with individual changes between +18% (H10R) and -72% (H2R). μ_2_ sunk by -46% on average, with individual changes between +48% (H10R) and -59% (H8L). The values of μ_1_ and μ_2_ decreased in all subjects, with the exception of H10R. μ_max_ decreased by -8% on average; it rose in seven subjects but sunk in three of them. The individual changes laid between +101% (H10R) and -42% (H9L).

A logarithmic correlation between the pOP month and μ was found for the ‘average’ subject, with R² = 0.992 (μ_1_), R² = 0.993 (μ_2_) and R² = 0.251 (μ_max_) (Table 5). Furthermore, the correlation was also calculated for each subject separately. The coefficients of correlation for μ_1_ ranged between 0.999 (H2R) and 0.226 (H10R)_,_ those for μ_2_ between 0.999 (H6R) and 0.745 (H8L) and for μ_max_ between 0.954 (H9L) and 0.007 (H1L), respectively.

## Discussion

In this study, it was possible for the first time to simultaneously measure joint contact forces and friction moments *in vivo* during walking. High friction moments may endanger the stability of cup fixation and cause fretting between the prosthesis head and the taper. Loosening moments of acetabular cups depend on the quality of the cup-bone interface, which is influenced, among others, by the fixation technique and the type of coating. For cementless press-fit cups, cyclic loosening moments as low as 8Nm were determine *in vitro* [35]. The maximal measured friction moment (intra-individual average!) was 0.402%BWm in H10R (Fig. 4), which corresponds to 3.99Nm or 50% of the critical value. This shows that the real friction moments, which act during higher demanding activities than walking or when extreme force occur during stumbling [36], may reach dangerous values, especially shortly after implantation.

### Joint loads and gliding parameters

Simulator tests on friction and wear in hip implants have been performed under varying conditions. In some cases, the joint was moved in one plane only, using sine wave loads and movements, while the movements around the other joint axes were ignored [11,18,21,37]. However, during walking, the hip joint moves not only around the x-axis (flexion/extension) but also around the y-axis (abduction/adduction) and the z-axis (internal/external rotation). An earlier study [30] showed that friction also depends on the moments around the other two axes. Furthermore, the joint movement is not sinusoidal, and the contact loads typically have two peaks at CTO and CHS. We have shown that the friction moment M_res_ increases *in vivo* between HS and CHS, when it reaches its maximum. This finding is contrary to the *in vitro* situation, where the measured friction moment showed a plateau phase during the loading of the joint [11,21].

In the current *in vivo* study, μ has been calculated in 3D and has been proven not to be constant throughout the gait cycle (Fig. 2). During each step it increased (Table 3, 4) from 0.045 to 0.175 (three months pOP) respectively from 0.028 to 0.179 (twelve months pOP). μ reaches its absolute maximum shortly before TO, when the movement changes from extension to flexion. At that instant, the relative velocity between ball and cup is either zero or very low. Earlier studies reported that μ differed when the lubrication conditions were changed [19,20,38]. The high peak value of μ is probably best explained by the assumption that the synovia is squeezed out under high loads and transported back into the joint when the load is low during the swing phase [21].

### Postoperative changes

No significant change of the resultant contact force F_res_ in the ‘average’ subject was observed between three and twelve months pOP. However, the joint loads during the first three months are not reported here. In an earlier study, F_res_ showed during that time an average increase of 18% (F_res1_) and 21% (F_res2_) when walking with crutches [39]. This data was only based on subjects H1L to H7R, who also participated in this study. Future simulations of bone remodeling around hip implants by finite element modeling should simulate increasing contact forces during the first three months while maintaining constant contact force values afterwards.

A significant decrease of the friction moment M_res_ in the postoperative course was observed throughout the whole gait cycle for the ‘average’ subject. At the instants of the two peaks of F_res_, M_res_ decreased significantly by 47% (M_res1_) and 34% (M_res2_). These changes may be caused by ‘running-in’ effects of the gliding components or by improved lubricating properties of the synovia [40]. A fluid with a high viscosity needs more time to flow out of the intra-articular joint space, provided that the contact force and sliding speed are the same. Synovia is a non-Newton fluid [40], and its viscosity therefore depends on contact force, sliding velocity and shear stress [13,19,40,41]. Because the walking velocity and contact forces were found to be nearly constant during the observed period, the decrease in M_res_ suggests an increase in fluid viscosity by about 50%, an effect which had not be investigated in the current study. The logarithmic decrease of the coefficient of friction over the postoperative time suggests a change in synovia viscosity and, possibly, an additional smoothening of the gliding surfaces.

Typical simulator studies, which investigated the friction of soft-on-hard parings as in our study (XPE/Al_2_O_3_), delivered μ-values between 0.04 and 0.09 [11,13,42] for a lubricated regime and for a ‘dry’ sliding condition values between 0.13 and 0.14 were determined [43]. The sharp increase of μ between CHS and TO did not change significantly between three and twelve months pOP. This finding suggests that during early flexion, hip joint friction does not depend much on synovial properties. This finding, along with the high absolute peak values μ_max_, indicates the presence of ‘dry’ friction [43] in the joint during the early flexion phase. However, several studies have shown that the fluid film thickness in a soft-on-hard pairing is much lower than the roughness of the polyethylene bearing surface [44,45]. This suggests that ‘mixed’ or ‘boundary lubrication’ conditions occurring for this tribological pairing [42].

A time-dependent logarithmic trend of μ_max_ was also observed, but only with R² = 0.251 and a high inter-individual range (0.013 to 0.886). The significance of postoperative changes of μ_max_ in only some of the subjects may be due to different activity levels and therefore different running in effects [46].

### Inter-individual differences

The observed peak values of F_res_ varied individually by 44% (three months pOP) and 34% (twelve months pOP). In contrast to these relatively small deviations, M_res_ individually differed by 129% (three months pOP) and even 451% (twelve months pOP). For μ, the individual variation laid between 141% (three months pOP) and 914% (twelve months pOP). These strong individual variations of M_res_ are most likely due to different ‘running-in’ effects, which are caused by the individual activity levels and synovial properties.

In subject H10R, the peak μ_max_ was extremely high and was greater than that observed in all of other subjects. This observation may possibly be explained if one assumes that in H10R the movement in the joint really fell to zero at the instant when the movement changed from extension to flexion. In the other subjects, however, the joint may still have been slightly rotating in the frontal and horizontal planes. The fluid film may thus have been broken down completely in the joint of H10R only. To clarify this hypothesis, future study will focus on individually different movements in the joint and on deviating areas of load transfer, which will be analyzed using individual gait data and anatomical conditions.

It was shown that the peak values of the *in vivo* acting friction moments during walking were smaller than critical torsion torque at the cup-bone interface [35]. However, higher friction moments can be expected during high demanding activities, which might be critical for the cup fixation, especially shortly after implantation.

This study has reported for the first time post operative changes and the individual differences of the *in vivo* forces and friction moments acting in total hip joint replacements. These in vivo loads can now be used as realistic input data for friction and wear simulator studies.

Examples of the *in vivo* measurements are published at the public data base www.orthoload.com.

### Limitations of the study

There are some limitations to this study. The number of investigated subjects was small and only one implant type was investigated only during level walking. The peak values of the friction moment and friction coefficient and their time-dependent changes individually varied extremely. Possible factors, causing these variations, were not evaluated. Such factors as implant orientation, sliding speed and contact area in the joint, as well as friction during other activities are currently investigated in an additional study.
